# Circadian rhythm of the autonomic nervous system in insulin resistant subjects with normoglycemia, impaired fasting glycemia, impaired glucose tolerance, type 2 diabetes mellitus

**DOI:** 10.1186/1471-2261-6-19

**Published:** 2006-05-02

**Authors:** Antonio Perciaccante, Alessandra Fiorentini, Alberto Paris, Pietro Serra, Luigi Tubani

**Affiliations:** 1 III Clinica Medica, Department of Clinical Medicine, University "La Sapienza", Rome, Italy; 2 Medicina Interna E, Department of Clinical Medicine, University "La Sapienza", Rome, Italy

## Abstract

**Background:**

In type 2 diabetes mellitus both insulin resistance and hyperglycemia are considered responsible for autonomic dysfunction. The relation between the autonomic activity, impaired fasting glycemia and impaired glucose tolerance is, however, unclear. The purpose of this study was to evaluate and compare the circadian autonomic activity expressed as heart rate variability (HRV) measured by 24-hours ECG recording in insulin resistant subjects (IR) with characteristics as follow: IR subjects with normal oral glucose tolerance test results, IR subjects with impaired fasting glucose, IR subjects with impaired glucose tolerance and subjects with type 2 diabetes mellitus.

**Methods:**

Eighty Caucasian insulin resistant subjects (IR) and twenty five control subjects were recruited for the study. IR subjects were divided into four groups according to the outcoming results of oral glucose tests (OGTTs): IR subjects with normal glucose regulation (NGR), IR subjects with impaired fasting glycemia (IFG), IR subjects with impaired glucose tolerance (IGT) and subjects with type 2 diabetes mellitus (DM). Autonomic nervous activity was studied by 24-hours ECG recording. Heart rate variability analysis was performed in time and frequency domains: SDNN, RMS-SD, low frequency (LF) and high frequency (HF) were calculated.

**Results:**

The total SDNN showed statistically significant reduction in all four groups with insulin resistant subjects (IR) when compared to the control group (p <0,001). During night LF normalized units (n.u.) were found to be higher in all four groups including IR subjects than in the control group (all p < 0,001) and subjects with normal glucose regulation (NGR), with impaired fasting glycemia (IFG) and with impaired glucose tolerance (IGT) were found to have higher LF n.u. than those in the type 2 diabetes mellitus group. The linear regression model demonstrated direct association between LF values and the homeostasis model assessment-index (HOMA-I), in the insulin resistant group (r = 0,715, p <0,0001).

**Conclusion:**

The results of our study suggest that insulin resistance might cause global autonomic dysfunction which increases along with worsening glucose metabolic impairment. The analysis of sympathetic and parasympathetic components and the sympathovagal balance demonstrated an association between insulin resistance and sympathetic over-activity, especially during night. The results indicated that the sympathetic over-activity is directly correlated to the grade of insulin resistance calculated according to the HOMA-I. Since increased sympathetic activity is related to major cardiovascular accidents, early diagnosis of all insulin resistant patients should be contemplated.

## Background

The sympathetic nervous system modulates both hepatic glucose production and the glucose uptake in peripheral tissues [1].

A relation between insulin resistance and cardiac autonomic regulation has been identified: some studies demonstrated that the increase of plasma insulin level was related to increased urinary [2] and plasma norepinephrine. Other studies, performed by microneurography, have shown that acute hyperinsulinemia increases sympathetic activity in muscle nerves [3].

Although insulin resistance predisposes to cardiovascular disease, its pathophysiology is poorly understood.

Analysis of heart rate variability (HRV), a non invasive technique used to assess autonomic neural regulation, has been successfully introduced to investigate the influence of autonomic neuromodulation in several pathophysiologic conditions [4-8].

Several studies performed by spectral analysis of HRV have shown sympathetic over-activity in insulin resistant subjects with normoglycemia [9-11].

The relation between diabetes mellitus and impaired cardiac autonomic activity has been identified: it is characterized by a reduced power in all spectral bands, impaired sympathetic response, abnormal reduced total power with unchanged low frequency/high frequency ratio (LF/HF ratio) [12,13].

The relation between to autonomic activity, impaired fasting glycemia and impaired glucose tolerance is, however, unclear.

Sigh et al [14] observed that the HRV was inversely related to plasma glucose levels: the total power reduction was greater in diabetic patients than that in subjects with impaired fasting glycemia. In Sigh's study the spectral analysis of HRV was performed only in 24-hours of ECG registration and the glucose metabolic impairment was calculated on fasting plasma glucose levels without previous oral glucose tolerance tests (OGTTs). Doing so, it's impossible to establish if some of these subjects with impaired fasting glycemia could also be affected by diabetes mellitus, identified by the criteria of The American Diabetes Association (ADA).

We hypothesized that insulin resistance might be related to sympathetic over-activity and that dysautonomia increases if insulin resistance is associated with glucose metabolic impairment.

To test this hypothesis we evaluated and compared the variations of the circadian autonomic rhythm, measured by means of heart rate variability (HRV) in insulin resistant subjects (IR) divided in four groups: IR subjects with normal glucose regulation (NGR), IR subjects with impaired fasting glucose (IFG), IR subjects with impaired glucose tolerance (IGT) and IR subjects with type 2 diabetes mellitus (DM). The analysis of these groups allowed us to demonstrate the dysfunction of autonomic system in insulin resistant subjects and to estimate the specific role of the progression of glucose metabolic impairment on autonomic activity.

## Methods

One hundred and fifty consecutive Caucasian subjects were screened. Oral glucose tolerance tests (OGTTs) were performed in all subjects after overnight fast. Blood samples for glucose and insulin tests were collected before and 2-hours after glucose load consisting in 75 g glucose anhydrate in 300 ml of water ingested over the course of 5 minutes. Moreover fasting plasma insulin levels were measured to evaluate the insulin resistance according to the homeostasis model assessment-index (HOMA-I). Subjects with hypertension [15], obesity, dyslipidemia, cardiac arrhythmias, microalbuminuria, other comorbidity (such as renal failure, heart failure, liver diseases, hypothyroidism and hyperthyroidism) and undergoing drug treatment that could potentially disturb carbohydrate metabolism (glucocorticoids, furosemide, B-blockers, etc) and cardiac autonomic activity (B-blockers, anti-arrhythmics, ACE-inhibitors) were excluded. Among them one hundred subjects (age 51,44 +/- 0,67 years, 51 men and 29 women) were admitted in this study: twenty subjects with normal OGTTs results and without IR (control group) and eighty with insulin resistance.

Height, weight and body circumferences were measured in all subjects; body mass index (BMI, kg/m^2^) was calculated as weight divided by height squared; waist-to-hip ratio (WHR) was defined as waist circumference divided by hip circumference.

The eighty patients with insulin resistance were divided into four groups following their OGTTs results as established b the criteria of The American Diabetes Association (ADA) [16]:

1) a group of subjects with normal glucose regulation (NGR): fasting plasma glucose (FPG) < 5,6 mmol/L and 2-hours plasma glucose (2-HPG) < 7,8 mmol/L.

2) a group of subjects with impaired fasting glycemia (IFG): F.P.G. 5,6 – 6,9 mmol/L and H.P.G < 7,8 mmol/L.

3) a group of subjects with impaired glucose tolerance (IGT): F.P.G < 7,0 mmol/L and 2-HPG 7,8–11.1 mmol/L.

4) a group of subjects with type 2 diabetes mellitus (DM): F.P.G. ≥ 7,0 mmol/L or 2-H.P.G. 7,8 – 11.1 mmol/L.

The glucose levels were determined by the glucose oxidative methods (Beckman Coulter, Inc., Fullerton, CA); the coefficient of variation for this assay was less than 4%. Insulin levels were measured in μU/ml by radioimmunoassay (Linco Research, Inc., St. Charles, MO). The lower limit for detection of insulin was 3 μU/ml. The intra- and extra-assay coefficients of variation were less than 4% and 10% respectively.

The control group consisted in sex and age matched healthy and normoglycemic subjects without insulin resistance.

Ethical approval was obtained from the Institutional Ethics Committee of the Umberto I° Hospital. Written informed consent was obtained from all participants; all the investigations were performed in accordance with the principles of the Declaration of Helsinki.

### Insulin resistance

The insulin resistance was evaluated by the homeostasis model assessment-index (HOMA-I) [17-19]. The HOMA-I was calculated by the formula: fasting plasma glucose (mmol/L) × fasting plasma insulin (μU/ml)/22,5 as described by Matthews and coworkers [20]. Insulin resistance was defined as the third and fourth quartiles of the HOMA-I. The accuracy and the precision of the HOMA methods have been compared to independent estimates of insulin resistance [20].

### HRV assessment

Autonomic nervous system function was evaluated by heart rate variability (HRV) analysis during 24-hours ECG recording. All Holter recording were performed using a three-channel recorder. Cardiovascular variability was analysed following the recommendations of the Task Force of the European Society of Cardiology and the North American Society of Pacing and Electrophysiology [21].

Spectral estimates of R-R intervals were obtained from stationary regions free of ectopic beats and technical artifacts. The standard deviation of normal-to-normal RR intervals [SDNN (ms) correlated to total autonomic activity] and the square root of the average of the sum of the squares of the differences between adjacent NN intervals [RMS-SD (ms) correlated to parasympathectic system] were calculated and were divided in two periods: night (0 a.m. – 6 a.m.) and day (7 a.m. – 9 p.m.). Fast Fourier Transform was used to obtain power spectral estimates of HRV, total power in the frequency range (0 – 0,40 Hz) was divided into: very low frequency (VLF: < 0,40 Hz), low frequency (LF: 0,04 – 0,15 Hz modulated by sympathetic system), and high frequency (HF: 0,15 – 0,40 Hz mediated by parasympathetic system), the integrals underlying respective power density function were measured and expressed in absolute units (ms^2^/Hz). Each spectral component was also presented in normalized units (n.u.) by dividing LF or HF by total power minus the very low frequency (LF or HF/total power – VLF). The LF/HF ratio, considered an index of cardiac sympathetic/parasympathetic tone balance, was also calculated.

Patients were analysed for 24-hours at 10 minutes intervals. Artificial data and arrhythmic events were excluded. The 24-hour period was divided into four parts: night (0 a.m. – 6 a.m.), morning (7 a.m. – 12 a.m.), afternoon (1 p.m. – 6 p.m.) and evening (7 p.m. – 11 p.m.). Data analyses were performed with Del Mar Avionics Accuplus 363, Irvine California, USA. In ten healthy subjects reproducibility was evaluated by means of the interclass correlation coefficient (ICC) comparing baseline values with the results obtained at the fourth week. ICC was > 0,7 for HRV.

### Statistical analysis

Statistical analysis was performed with SPSS 12,0 (SPSS Inc., Chicago, IL, U.S.A.) for Windows XP. Normality tests were performed on all data. Parametric data are expressed as mean values +/- standard deviation (SD) or data with multiple time points variables were analysed by the general model ANOVA. *Post hoc *multiple comparisons were performed using LSD test when ANOVA testing was significant (p < 0,05). Pearson correlation coefficients were computes to qualify the relationship between the variables. Since the levels of the HOMA-I strictly depended on fasting insulin and glucose concentration, we did not consider these parameters in multiple regression analysis. P < 0,05 was considered statistically significant.

## Results and discussion

### Clinical characteristics

Clinical characteristics for each study-group are shown in Table 1.

**Table 1:** Clinical characteristics for each study-group of subjects with insulin resistance and control group.

	NGT	**IFG**	**IGT**	**DM**	**Controls**	**p (ANOVA)**
**Sex (M/F)**	12/8	14/6	12/8	13/7	15/5	
**Age (years)**	49,1 ± 6,6	49,8 ± 6,5	53,6 ± 3,7	52,4 ± 4,7	53,0 ± 8,0	NS
**BMI (kg/m**^2^**)**	27,3 ± 4,9	28,1 ± 4,7	26,2 ± 3,01	27,3 ± 2,8	25,0 ± 5,1	NS
**WHR**	0,97 ± 0,07	0,96 ± 0,04	0,98 ± 0,06	0,97 ± 0,05	0,9 ± 0,06	NS
**Fasting plasma insulin (uU/ml)**	19,3 ± 4,3	10,9 ± 2,9	12.67 ± 2.14	10.2 ± 2.9	7,1 ± 0,2	<0.001
**HOMA-I**	4,4 ± 0,8	3,1 ± 0,8	3,1 ± 0,6	2,7 ± 0,4	1,4 ± 0,2	
**HR**	74,7 ± 4,5	76,6 ± 10,6	76,7 ± 10,9	75,0 ± 12,7	70,2 ± 4,1	NS
**SBP (mmHg)**	123,2 ± 3,7	121,1 ± 5,3	117,3 ± 11,2	121,0 ± 6,3	120,2 ± 7,0	NS
**DBP(mmHg)**	80,0 ± 3,1	78,1 ± 4,0	77,1 ± 6,7	77,7 ± 3,8	78,8 ± 4,9	NS

Values are mean ± SD. P <0.05 was chosen to be the threshold of statistical significance for ANOVA. BMI: body mass index, HOMA-I: the homeostasis model assessment-index, WHR: waist to hip ratio, SBP: systolic blood pressure, DBP: diastolic blood pressure, HR: heart rate; NGR: normal glucose regulation, IFG: impaired fasting glycemia, IGT: impaired glucose tolerance, DM: type 2 diabetes.

The groups were matched according to age, sex, anthropometrical parameters (i.e. body mass index, waist and hip circumferences, waist ratio) and smoking status.

### Heart rate variability

#### Time domain

Table 2 shows the comparison of the autonomic measures in time domain analysis of heart rate variability (HRV) between the different groups. The total SDNN showed statistically significant reduction in the insulin resistant groups when compared to the control group (p <0,001). DM group had smaller SDNN than the NGR group and the IFG group (p < 0,001, p:0,010, respectively). No significant difference between the DM and the IGT groups (p: 0,202) was identified. During night SDNN was significantly higher (all p < 0,001) in the NGR group and in the control group than in the IFG, IGT and DM groups. In all four groups with insulin resistant subjects, these results showed a reduction of total autonomic activity.

**Table 2:** Time domain measures for each insulin resistant group and control group.

	**NGR**	IFG	**IGT**	**DM**	**Controls**	P (ANOVA)
**SDNN (ms)**	130,9 ± 35,9*	117,9 ± 24,2*	103,1 ± 37,0*,**	89,6 ± 30,3*,**	176,6 ± 29,5	< 0,001
**SDNN day (ms)**	105,2 ± 28,3	109,4 ± 31,7	81,1 ± 18,6*,**	77,3 ± 23,5 *,**	120,2 ± 26,9	< 0,001
**SDNN night (ms)**	118,8 ± 46,1	71,9 ± 11,8*,**	77,2 ± 32,0*,**	69,0 ± 22,1*,**	119,5 ± 38,2	< 0,001
**RMS-SD (msec)**	43,15 ± 20,33	35,16 ± 10,06	27,19 ± 12,46**	32,78 ± 15,51**	36,75 ± 10,59	0,032
**RMS-SD day (msec)**	41,58 ± 18,14*	34,46 ± 10,47	23,93 ± 11,41**	32,34 ± 14,46**	31,37 ± 9,07	0,007
**RMS-SD night (msec)**	47,47 ± 29,26	36,78 ± 11,01*	30,76 ± 15,34*,**	32,43 ± 15,51*,**	47,47 ± 17,60	0,013

Results are expressed as mean ± SD for each group. P < .05 was chosen to be the threshold of statistical significance for ANOVA and LSD *post hoc *test. SDNN: standard deviation of all sinus rhythm RR intervals ms, RMS SD: the square root of the mean of the sum of the squares of differences between adjacent NN intervals, NGR: normal glucose regulation, IFG: impaired fasting glycemia, IGT: impaired glucose tolerance, DM: type 2 diabetes.

* P < .01 for comparison with control group.

** P < .05 for comparison with NGR grou

There was no difference in RMS-SD in insulin resistant subjects during night.

### Frequency domain

The measures of total power in frequency domain analysis of HRV are resumed in Table 3. The total power was statistically reduced in the insulin resistant groups (p < 0.001). No significant difference between the NGR. and the IFG groups (p: 0.552) was identified. The IGT group showed smaller total power than the IFG group (p: 0.002).

**Table 3:** Autonomic function measures expressed in absolute values for each insulin resistance group and control group

	NGR	**IFG**	**IGT**	**DM**	**Controls**	**p (ANOVA)**	
Total power (msec^2^/Hz)	2263,9 ± 312,4*	2135,2 ± 422,5*	1432,3 ± 36,7*	317,3 ± 96,7*	3798,6 ± 758,6	< 0,001	
LF (msec^2^/Hz)	332,1 ± 66,6*	258,3 ± 12,8*	236,11 ± 5,5*	18,6 ± 3,1*	692,8 ± 202,7	< 0,001	
HF (msec^2^/Hz)	85,5 ± 36,1	134,4 ± 24,4	93,6 ± 1,2	17,9 ± 3,4	446,8 ± 102,4	< 0,001	
R-R (ms)	936,1 ± 10,3	886,8 ± 36,5	888,2 ± 13,1	788,5 ± 100,4	983,2 ± 182,4	0,005	
**Night**							
Total power (msec^2^/Hz)	2705,7 ± 360,7*	1059,9 ± 85,2*	1187,8 ± 81,7*	338,6 ± 177,4*	4590,8 ± 332,6	< 0,001	
LF (msec^2^/Hz)	992,2 ± 7,2	209,6 ± 19,3*,**	563,2 ± 12,1*,**	32,09 ± 0,8*,**	876,7 ± 343,1	< 0,001	
HF (msec^2^/Hz)	320,4 ± 141,7	179,4 ± 106,1	145,2 ± 52,9	54,5 ± 22,6	1412,3 ± 731,6	< 0,001	
R-R (ms)	1072,2 ± 36,0*	918,1 ± 127,2*	877,0 ± 125,4*	869,6 ± 73,5*	131,7 ± 148,1	< 0,001	
**Morning**							
Total power (msec^2^/Hz)	2068 ± 931,6	1944,8 ± 482,8*	1355,0 ± 824,8*	92,7 ± 11,9***	3121,8 ± 683,0	< 0,001	
LF (msec^2^/Hz)	701,7 ± 155,0	1210,2 ± 556,9*,**	470,6 ± 181,5	40,0 ± 29,8*	1286,2 ± 420,7	< 0,001	
HF (msec^2^/Hz)	245,8 ± 222,6	506,5 ± 66,0	236,4 ± 173,5	5,5 ± 0,2	605,3 ± 246,2	< 0,001	
R-R (ms)	868,4 ± 106,5	932,8 ± 67,8	831,2 ± 92,3	753,1 ± 200,1	863,9 ± 216,0	NS	
**Afternoon**							
Total power (msec^2^/Hz)	2152,4 ± 500,1	1982,6 ± 1251,0	1446,9 ± 90,2***	675,8 ± 577,2*,**	2677,7 ± 1445,2	0,002	
LF (msec^2^/Hz)	969,1 ± 658,4	640,7 ± 395,6	487,3 ± 76,5***	69,3 ± 38,7*	898,6 ± 166,8	< 0,001	
HF (msec^2^/Hz)	176,6 ± 84,7	231,0 ± 143,5	118,4 ± 13,6	58,9 ± 27,1	296,2 ± 90,8	<0,001	
R-R (ms)	865,7 ± 50,0	849,0 ± 0,8	887,5 ± 131,2	793,8 ± 103,8	874,0 ± 185,7	NS	
**Evening**							
Total power (msec^2^/Hz)	1588,1 ± 756,9*	1452,2 ± 71,7*	1841,7 ± 1024,3*	111,8 ± 7,8*,**	4032,3 ± 2296,4	< 0,001	
LF (msec^2^/Hz)	728,4 ± 403,7***	360,9 ± 54,6*	725,1 ± 358,7***	41,6 ± 14,4*	1382,8 ± 1266,9	< 0,002	
HF (msec^2^/Hz)	180, 2 ± 31,6	262,1 ± 171,0	394,7 ± 239,5	16,9 ± 7,4	775,9 ± 492,3	< 0,001	
R-R (ms)	891,4 ± 29,1*	814,8 ± 85,1*	939,6 ± 36,0	743,7 ± 18,6*	1039,2 ± 215,0	< 0,001	

Values are mean ± SD for each group, P <,05 was chosen to be the threshold of statistical significance for ANOVA and LSD *post hoc *test, NGR: normal glucose regulation, IFG: impaired fasting glycemia, IGT: impaired glucose tolerance, DM: type 2 diabetes, LF: low frequency, HF: high frequency, hours of Holter registration: night time = 0–6 am, morning = 7–12 am, afternoon = 1–6 pm, evening = 7–11 pm, LF/HF: low frequency/high frequency.

* P <,01 for comparison with control group.

** P <,05 for comparison with NGR group.

*** p <,05 for comparison with controls.

The normalized units (n.u.) are described in Table 4. The total LF were higher (all p < 0.001) in the NGR, the IFG and the IGT groups than in the DM and in the control groups. LF were higher in the NGR group when compared to the IFG group (p: 0.043) and there was no difference between the DM group and the control group (p: 0.114), between the IFG and the IGT groups (p: 0.077) and between the NGR and the IGT groups (p: 0.828). HF were lower in the NGR group (p < 0.001), in the IGT group (p < 0,001) and in the DM group (p:0.02) than in the control group. HF were lower in the NGR and in the IGT groups than in the IFG group (p: 0.02, p: 0.04) and in the DM group (p <0.001). There were no differences between the NGR and the IGT groups and between the IFG and the DM groups.

**Table 4:** Autonomic function measures, expressed in normalized units, for each insulin resistance group and controls.

	**NGR**	**IFG**	**IGT**	**DM**	**Controls**	P (ANOVA)
**LF n.u.**	70,1 ± 6,6*	65,7 ± 8,2*,**	70,3 ± 5,8*	59,0 ± 7,3	55,4 ± 7,5	0,000
**HF n.u.**	24,3 ± 6,5	28,5 ± 7,4	23,5 ± 6,5	32,6 ± 6,4	37,6 ± 7,5	0,000
**LF/HF **	3,1 ± 1,3	2,5 ± 0,8	3,3 ± 1,4	1,9 ± 0,5	1,4 ± 0,9	0,000
**LF night n.u.**	66,7 ± 9,8*	65,5 ± 8,4*	69,8 ± 5,3*	54,8 ± 11,9*	35,2 ± 5,9	0,000
**HF night n.u.**	26,7 ± 8,1	29,1 ± 6,5	24,9 ± 6,0	37,8 ± 10,9	58,6 ± 6,6	0,000
**LF/HF night**	2,9 ± 1,5	2,4 ± 0,9	3,0 ± 0,9	1,6 ± 0,7	0,7 ± 0,5	0,000
**LF n.u. morning**	72,4 ± 9,4	63,5 ± 9,2	69,5 ± 7,2	59,4 ± 6,6*	69,4 ± 8,4	0,000
**HF n.u. morning**	22,7 ± 7,1	28,6 ± 8,7	22,8 ± 7,9	34,1 ± 5,9	23,0 ± 7,1	0,000
**LF/HF morning**	3,9 ± 2,8	2,5 ± 1,4	3,6 ± 1,8	1,8 ± 0,5	3,6 ± 1,7	0,001
**LF n.u. afternoon**	69,2 ± 9,8	70,3 ± 12,1	72,1 ± 7,3	50,4 ± 7,2*	70,0 ± 6,6	0,000
**HF n.u. afternoon**	24,1 ± 9,8	27,3 ± 14,9	21,3 ± 8,0	34,6 ± 9,4	22,3 ± 5,1	0,002
**LF/HF afternoon**	4,0 ± 3,9	3,7 ± 2,6	4,1 ± 2,3	1,6 ± 0,7	3,6 ± 1,3	0,028
**LF n.u. evening**	72,1 ± 6,3*	64,9 ± 15,3***	69,0 ± 7,9***	59,6 ± 5,9	56,7 ± 7,7	0,000
**HF n.u. evening**	23,6 ± 6,1	27,6 ± 14,6	25,3 ± 6,7	33,2 ± 6,3	35,7 ± 9,1	0,001
**LF/HF evening**	3,2 ± 0,9	3,6 ± 3,2	3,0 ± 1,4	1,8 ± 0,5	2,0 ± 0,9	0,008

Values are mean ± SD for each group. P < .05 was chosen to be the threshold of statistical significance for ANOVA and LSD *post hoc *test. NGR: normal glucose regulation, IFG: impaired fasting glycemia, IGT: impaired glucose tolerance, DM: type 2 diabetes, LF: low frequency, HF: high frequency, hours of Holter registration: night time = 0–6 am, morning = 7–12 am, afternoon = 1–6 pm, evening = 7–11 pm, LF/HF: low frequency/high frequency.

* P < .01 for comparison with control group.

** P < .05 for comparison with NGR group.

*** p < .05 for comparison with controls.

### Night (0 am – 6 am)

The insulin-resistant groups showed, in total power, statistically significant reduction when compared to the control group (all p < 0.001).

The total power showed statistically significant reduction in IR groups significant reduction

**Figure 1:**
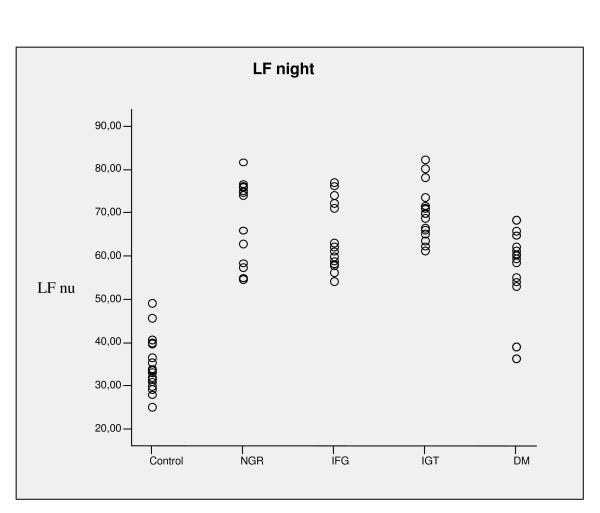
**Low frequency values during night in insulin resistant groups and controls**. LF: low frequency, NGR: normal glucose tolerance, IFG: impaired fasting glycemia, IGT: impaired glucose tolerance, DM: type 2 diabetes.

LF were higher in the four groups with IR than in the control group (all p < 0.001) and in the NGR, IFG and IGT groups when compared to the DM group [fig. 1]. HF were lower in all insulin-resistant groups than in the control group (all p < 0.001), and there were no differences among the NGR, IFG and IGT groups.

In all insulin-resistant groups LF were higher than in the control group (all p < 0.001), and in subjects with NGR, IFG and IGT LF were increased than in the DM group [fig. 1]; HF were lower in all insulin-resistant groups than in the control group (all p < 0.001), and among the NGR, IFG and IGT groups the differences were not statistically significant.

### Morning (7 am – 12 am)

In total power during the morning significant differences were no identified between the NGR group and the control group (p: 0.131). Normalized LF were not statistically differet among in the NGR, the IGT and the control groups and between the IFG and the DM groups (p: 0.140), but LF were higher in the NGR and the IGT groups than in the IFG (p: 0.001, p: 0.046 respectively) and the DM (all p < 0.001) groups. LF were lower in the IFG and the DM groups than in the control group (p: 0.038, p < 0.001 respectively).

HF were reduced in the NGR and the IGT groups when compared (with) to the IFG (p: 0.015, p: 0.031 respectively) and to the DM groups (all p < 0.001) and in the IFG group than in the DM group (p: 0.028). HF were higher in the IFG and the DM groups than in the control group (p: 0.029, p < 0.001, respectively). The HF values were not statistically different among the NGR, the IGT and the control groups.

### Afternoon (1 pm – 6 pm)

The total power in NGR and IFG was not statistically different versus control group (p: 0.258 and p: 1.37 respectively). LF were lower in the DM group than in the other groups (all p < 0.001) and HF were higher in the DM group than in the NGR (p: 0.001), IGT (p < 0.001) and control groups (p: 0.004). The LF and HF values were not different among the NGR, IGT and control group.

### Evening (7 p.m. – 11 p.m.)

The total power was statistically different in the NGR, the IGF and the IGT groups when compared to that of the control and the DM groups. LF were higher in the NGR and in the IGT groups than in the DM group (all p < 0.001) and those in the control group (p < 0.001, p:0.002, respectively), and LF values were higher in the IFG group than in those in the control group (p:0.034), and there was no significant difference between the NGR and the IGT groups, between the IFG and the DM groups and between the DM group and the control group.

The frequency domain analysis of HRV showed association between insulin resistance and sympathetic over-activity especially during night.

**Figure 2:**
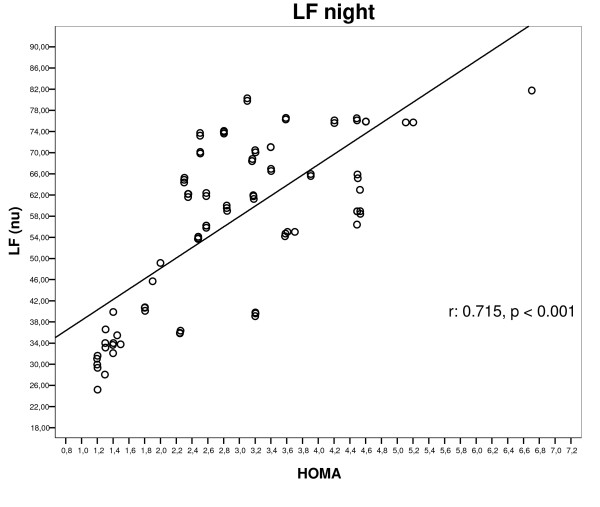
**Correlation between low frequency (LF) during night and HOMA. **LF: low frequency, HOMA-I: homeostasis model assessment-index. r = 0.715, p < 0.0001

As demonstrated in figure 2 linear regression model showed direct association between LF value and the HOMA-I, in the insulin resistant group (r = 0,715, p < 0.0001).

A limit in this study is the use of the HOMA-index as a conventional indicator of insulin resistance. The best method for assessing insulin resistance is the glucose clamp technique. However the HOMA model has proved to be a robust clinical and epidemiological tool in description of the pathophysiology of diabetes and, as already quoted in over 500 publications, it has become one of the standard tools in the *armamentarium *of the clinical physiologist [23].

## Conclusion

Our study evaluates and compares the circadian autonomic rhythm measured by heart rate variability (HRV), obtained by 24-hours ECG Holter registration in insulin resistant subjects with normal oral glucose tolerance test, with impaired fasting glucose, with impaired glucose tolerance and with type 2 diabetes mellitus. The results of the present study confirm that impaired autonomic activity was present also in insulin resistant patients with normal glucose metabolism and in those with impaired glucose tolerance.

We demonstrated that the sympathovagal balance (expressed by the LF/HF ratio) remains consistently altered with a sympathetic over-activity during night in all insulin resistant subjects. This altered balance is revealed by the lack of increased parasympathetic component (HF n.u.) and the lack of reduction in the sympathetic component (LF n.u.).

**Figure 3:**
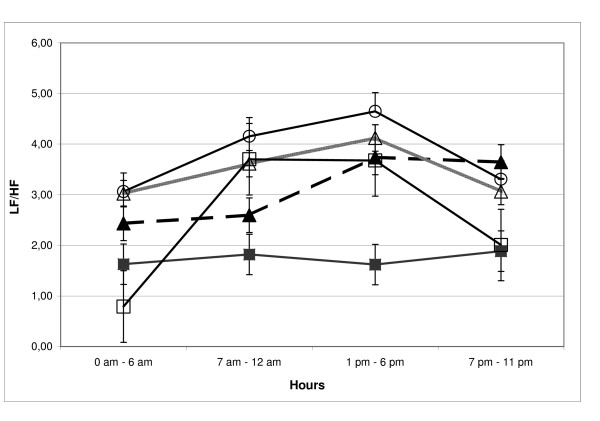
**Circadian variation of LF/HF in insulin resistant subjects and in control group. **White circles: groups with normal glucose tolerance; white triangles: groups with impaired glucose tolerance; black triangles: groups with impaired fasting glycemia, black squares: groups with type 2 diabetes; white squares: controls, LF/HF: low frequency/high frequency.

The sympathetic over-activity is mainly shown in the non diabetic insulin resistant groups rather than in the diabetic group: in fact in this group the circadian rhythm of autonomic activity showed no changes as demonstrated by the sympathovagal balance that remained stabile during both night and day [fig. 3].

These observations showed that autonomic dysfunction is present in all insulin resistant groups, but the subjects with type 2 diabetes mellitus had greater autonomic dysfunction than the insulin resistant subjects in the NGR, the IFG and the IGT groups had.

Moreover this study claims that the autonomic dysfunction (AND) is linearly related to insulin resistance. The relationship between the sympathetic over-activity and the increase of insulin resistance, calculated by HOMA-Index, is showed in figure 2. Increase of HOMA-I value was correlated to increase of the LF values (r = -0.718, p <0.0001).

**Figure 4:**
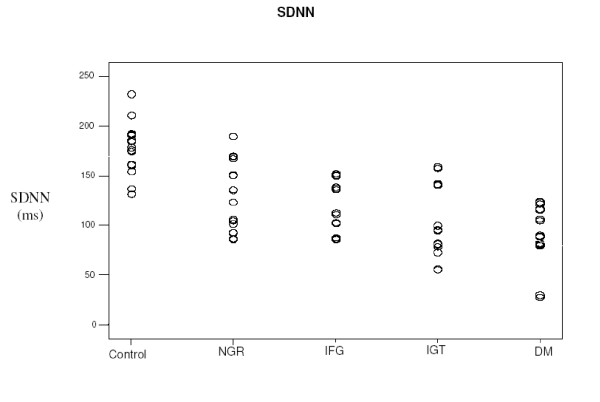
**SDNN values in insulin resistant groups and controls. **SDNN: standard deviation of all sinus rhythm RR intervals ms, NGR: normal glucose tolerance, IFG: impaired fasting glycemia, IGT: impaired glucose tolerance, DM: type 2 diabetes. NGR, IFG, IGT, DM vs. control: p < 0.001, NGR, IFG vs. DM: p < 0.001, IGT vs. DM: p: 0.202.

In the time domain analysis of HRV our study demonstrated significant reduction of the total autonomic system activity in all insulin resistant groups, expressed as progressive decrease of the SDNN values from the NGR the IFG, the IGT to the DM groups [fig. 4].

The data support the hypothesis that insulin resistance might cause a global reduction of the autonomic nervous system activity. Our study also demonstrated that the dysautonomia increases if insulin resistance is associated with worsening glucose metabolic impairment. In the NGR group the dysautonomia is due to the effect of hyperinsulinemia, whereas in the IFG and in the IGT groups we have found that the effects of moderate hyperinsulinemia and of moderate hyperglycemia is directly implicated in the dysautonomia.

Considering that sympathetic over-activity is related to major cardiovascular accidents, early diagnosis and treatment of all insulin resistant patients should be considered, especially in patients with sympathetic over-activity and with I.F.G. and/or I.G.T nowadays called "pre-diabetes".

We hypothesize that early treatment of insulin resistance might determine reduction in dysautonomia and consequently determine reduction of the risk of cardiovascular mortality risk.

Other studies are necessary to determine the mechanism whereby insulin resistance might be related to autonomic dysfunction.

## Abbreviations

**BMI**: body mass index; **DM**: type 2 diabetes mellitus; **HF**: high frequency; **HOMA-I**: the homeostasis model assessment-index; **HRV**: heart rate variability; **IFG**: impaired fasting glycemia; **IGT**: impaired glucose tolerance; **LF**: low frequency; **NGR**: normal glucose regulation subjects; **OGTTs**: oral glucose tolerance tests; **RMS-SD**: the square root of the mean of the sum of the squares of differences between adjacent NN intervals; **SDNN**: The standard deviation of normal-to-normal RR intervals; **WHR**: waist-to-hip ratio.

## Competing interests

The author(s) declare that they have no competing interests.

## Authors' contributions

AP conceived the study and participated in its design and coordination; AF participated in the design of the study and performed the statistical analyses; AP^1^, PS and LT participated in the design of the study and drafted the manuscript.

All authors read and approved the final manuscript

AP^1^:Alberto Paris

## Pre-publication history

The pre-publication history for this paper can be accessed here:


